# “Death drive” scientifically reconsidered: Not a drive but a collection of trauma-induced auto-addictive diseases

**DOI:** 10.3389/fpsyg.2022.941328

**Published:** 2022-09-28

**Authors:** Michael Kirsch, Aleksandar Dimitrijevic, Michael B. Buchholz

**Affiliations:** ^1^Institute of Physiological Chemistry, University Hospital Essen, Essen, Germany; ^2^International Psychoanalytic University, Berlin, Germany

**Keywords:** drive theory, death drive, trauma, masochism, auto-addictive disorders, biochemical analysis

## Abstract

Over the last 102 years, a lot of discussion was being held about the psychoanalytic conception of the “death drive,” but still with inconclusive results. In this paper, we start with a brief review of Freud’s conception, followed by a comprised overview of its subsequent support or criticisms. The core of our argument is a systematic review of current biochemical research about two proposed manifestations of the “death drive,” which could hopefully move the discussion to the realm of science. It was already established that drive satisfaction leads to the secretion of beta-endorphins, and research evidence also shows that the same biochemical mechanisms get activated in the case of masochism and the gambling disorder but only if they are preceded by chronic frustration of the essential drives. We conclude that the actual situation is more complex than Freud hypothesized, and that a fundamental revision of the psychoanalytic drive theory is necessary.

## Introduction

### Freud’s conception of the death drive

Initially, the idea of a death drive was proposed by others and Freud did not show much interest. It was first discussed in Freud’s house on 24 April 1907, and Alfred Adler was probably the first to write a paper about the “aggression drive” (Adler, 1908). More substantial was the paper by Sabrina Spielrein, “Destruction as the Cause of Coming Into Being” (Spielrein, 1913), which most probably influenced Freud several years after its publication. Ferenczi had ideas of his own, but for a time Freud was not interested: “I developed my philosophical views in front of Lou Salomé, which more or less corresponded to those in “Beyond,” although they end up somewhat differently. I also spoke to Freud about these views on occasion”^1^ [Ferenczi to Groddeck, 25 December 1921; (Fortune, 2002), p. 10; Avello, 2018].

The World War I provided the context, if not indeed the cause, for Freud’s first essay about the “death drive,” and in two different ways. Freud noticed during the Fifth psychoanalytic congress, held in Budapest in September 1918, that there were fundamental problems in both psychoanalytic practice and theory: recurring nightmares of the “Shell Shock” patients were inexplicable within the bounds of what he proposed in “The Interpretation of Dreams.” If dreams were coded wish fulfillments, what was the secret desire behind battle massacre scenes many dreamt about every night? This problem combined with Freud’s long-term efforts to develop a model of the mind based on a fundamental conflict, in which there would be only two drives and the sexual drive would have a worthy opponent for the perpetual struggle.

In a series of publications, Freud then introduced the idea of a “death drive,” “an innate, independent, instinctual disposition” (Freud, 1930, p. 102), a mysterious force connected to cosmology as much as to the human aggression,^2^ and mostly manifested in the repetition compulsion and the hypothesized wish of all life to return to inactivity and death. Freud also believed that this “death drive” was powerful enough to oppose the sexual drive and form the origin of inner conflict, so that the two of them operate against each other (Freud, 1940, p. 71). Freud (1923) clarified that three types of motivational drives (sexual drive, thirst, and hunger) are constituent elements of what he called *Eros*: “According to this view we have distinguished two classes of instincts, one of which, the sexual instinct or *Eros*, is by far the more conspicuous and accessible to study. It comprises not merely the uninhibited sexual instinct proper and the instinctual impulses of an aim-inhibited or sublimated nature derived from it, but also the self-preservative instinct…” (p. 40). It remains unclear, though, what the basis for these claims was.

Psychoanalysts of future generations who consider themselves Freudians still consider the “death drive” as a relevant idea. This pertains mostly to Melanie Klein and Lacan, and the subsequent generations of Kleinians, including Hanna Segal and Otto Kernberg, and contemporary Lacanians. Over time, the “death drive” turned from a philosophical conception to a supposedly clinical fact and influenced (especially Kleinian) modes of working with patients in each individual session.

### Criticism of the death drive from the viewpoint of the trauma theory

In the beginning, Freud treated all these ideas very tentatively (Freud to Ferenczi, March 28 and 31, 1919), and as late as 1935 he wrote, “Naturally, all this is groping speculation, until one has something better” (Freud to Jones, 3 March 1935).

Many of Freud’s closest collaborators, like Abraham, Jones, or Glover, did not subscribe to the “death drive” idea. In his final papers, Ferenczi tried to re-introduce the significance of trauma, which he saw as fundamentally connected to death not as a source but as an outcome: “trauma is a process of dissolution that moves toward total dissolution, that is to say, death” (Ferenczi, 1995, p.130), so that finally splitting “leads us too close to the problem of death!?” (Ferenczi, 1995, p. 41). At moments, it seems like you are reading Melanie Klein, except for one word: “Is not anxiety therefore in the last analysis a feeling of the power of the death drive, a beginning of death (starvation)?” (Ferenczi, 1995, p. 115). Ferenczi anticipates Klein’s opinion that anxiety is caused by the intensity of the death drive, but parenthetically adds that the drive itself is caused by the intensity of actual frustration – starvation. He provides a list of the consequences of this death wish caused by the lack of love (Ferenczi, 1995): “Moral and philosophic pessimism, skepticism, and mistrust became conspicuous character-traits in these patients […] repugnance to work, incapacity for prolonged effort, and thus a certain degree of emotional infantilism, naturally not without attempts at forced character-strengthening […] all the ‘unwelcome children’ of the male sex observed by me suffered from more or less severe disturbances of potency” (pp. 126–127). Ferenczi wrote even more explicitly against the idea of the “death drive” in several unpublished manuscripts (Ferenczi, 1929). On 13 August 1932, he notes down in his diary that “the idea of the death instinct goes too far, is already tinged with sadism” (Ferenczi, 1995, p. 200), and also calls it a pessimistic mistake (Ferenczi, 1930-32).

This led to a different brand of psychoanalysis (Dimitrijević et al., 2018), which persons like Ernest Jones and Freud himself were not ready to accept. So important were the ideas of Oedipus complex and the “death drive” that Sandor Ferenczi’s late work was censored between his death in 1933 and 1985, and even many of his analysands, like Melanie Klein, quoted him only rarely, if at all, and we can only find him in the subject indexes of their books.

Through his translator Jane Isabel Suttie, however, and through his assistant and literary executor Michael Balint, Ferenczi’s thinking became the foundation of the trauma theory in psychoanalysis and beyond. Suttie (1935), Jane’s husband, must have been familiar with Ferenczi’s work when he broke the self-imposed “taboo on tenderness” in psychoanalysis. The ripple reached John Bowlby, who among other things was the most important psychoanalytic Darwinian and a naturalist. With the help of ethology, it became clear to Bowlby (1969, 1973a,b, 1980) that nature prepares us to fight off death threats as long as we need to procreate and protect our offspring. In the US, different guidelines for psychoanalytic approaches to the “death drive” were developed by Fromm (1973), who introduced social and cultural factors along the hypothesized biological ones, and Kohut (1972), who advocated the primacy of the self and saw drives as a by-product of the, more or less temporary, dissolution of the self.

### “Death drive” in contemporary psychoanalysis

Since then, more than a hundred papers debating the “death drive” were published. Kernberg (2009) attempted to integrate diverse psychoanalytic concepts, but ended the paper cryptically:

The death drive, I propose, is not a primary drive, but represents a significant complication of aggression as a major motivational system, is central in the therapeutic work with severe psychopathology, and as such is eminently useful as a concept in the clinical realm. (p. 1018).

The only point that remains certain is that nothing gets clearer after this. If the “death drive” is not a primary drive, why is it still called a drive? One would assume that Kernberg will arrive at another conclusion, but he terms “useful as a concept” something that has never been clearly defined. Indeed, it seems the concept is useful as a projection device for clinicians who face a patient’s aggression.

More recently, the debate was summarized in a very accessible fashion by Karbelnig (2021), who has concluded that it includes frequent repetitions and is oriented toward prominent names, depending on the author’s belief systems and clinical identity, and suggests that we should “lay the death drive to rest.”

Revisions of the drive theory, especially in the case of “death drive,” have taken up a lot of points, as was recently coherently demonstrated by Solms (2021), who delivered a careful review of Freudian theoretical positions and showed how Freud fought against the too narrow biological assumptions of his time. This led Freud to the false conclusion that drive satisfaction is not ruled by the Nirvana principle, satisfaction or, as Solms proposes, “satiation of a drive,” but that it is deadly. The truth is just the opposite:

…satiation or “nothingness” (i.e., no needs making demands on the mind for work) is the ideal state of all self-preservative and libidinal creatures: it represents the successful outcome of work performed in response to drive demands. … Therefore, there is no need to invoke the existence of a separate “death” drive that serves the Nirvana principle; it is served by the “life” drives and it represents their ideal state. To be clear, there is no need (or justification) to conclude that “the aim of all life is death” (Freud, 1920, p. 38; his emphasis). Here, again, Freud was conflating psychology with biology. (Solms, 2021, p. 1054).

This intermingling of biological and psychological approaches is, indeed, confusing. We do not have sex in order to fulfill our biological task of reproducing humankind. This is the biological perspective; psychologically, however, we have sex because of the exchange of lust and of attachment needs. Binding these two drives together is the task of another motivational entity, playfulness.^3^ So it is a fundamental error to assume that satisfaction is related to the “death drive” or the Nirvana principle.

The clinical pathological manifestations, which Freud wanted to better understand by conceptualizing the “death drive,” like narcissism, addictions, homicidality, or suicidality, are attempts at achieving satiation by “short circuit”:

…that is, they are attempts to satisfy the demands made upon the mind for work without actually doing the work. In other words, they are attempts to evade the reality principle, which is indeed a dangerous (and potentially fatal) thing to do. These are failures of ego functioning. (Solms, 2021, p. 1054).

Over many years the concept of a “death drive” was also used to “explain” human violence in all of its varieties. It was meant to explain violence in marriages, warfare, sexual abuse, and even terrorism (for a critical overview see Akhtar, 2017). The “death drive” seemed to be the thread connecting all these fields with clinical diagnoses of various kinds (paranoia, mistrust, Obsessive–Compulsive Disorders, personality disorders, phenomena of splitting). However, there are several serious logical inconsistencies here.

### Logical fallacies in the “death drive” idea

Drives, Freud postulated, had four distinct elements: source, pressure, aim, and object. From somewhere in our organisms an unpleasant sensation comes, either that there is too little or too much of something in us, and we feel an urge to find whatever will help us return to the calmness of the beginning level and enjoy satisfaction, until the drive wish returns and grows in intensity. Most people need several meals a day, one dies without water within a couple of days, many young couples enjoy sex so frequently that their abdominal muscles get sore. If the “death drive” is a drive, should it not work the same way? If it does not work like other drives, why name it a drive and place it in the same category?

There are also several problems when it comes to the explanation of violence (Aho, 2013) with the use of “death drive.”

First, if you explain *disorders* by “death drive” you cannot use in the same move that concept to explain human *order*. Freud’s concept of the “death drive” is not only described as anthropologically universal but it reaches far beyond into the cosmological. But you are then obliged to explain why some people are dis-ordered and the others are not.

Second, extending the concept so far into the anthropological universalism provides everybody with a universal excuse: “It’s not me, it’s the drive!” Pretexts of this kind have been found in studies on rape where perpetrators excuse themselves by the “irresistible” attraction of the victim (Beneke, 1982; Drew, 1992; Newcombe et al., 2008; Ryan, 2011). The assumption of an “irresistible” drive operates as justification; but it, nevertheless, does not explain why a wide majority of men do not rape women, although they often use similar kinds of rape myths when they talk about rapists (Edwards et al., 2011).

Third, the assumption of a “death drive” does neither explain nor excuse anything in sexual or in violent affairs. Freud maintained that only the Ego has access to (social) motor skills. If this holds true, ethical responsibility enters theory. Turned into epistemological terms, the drive is the equivalent of determinism, while the Ego is the equivalent of choice. As the concept of “death drive” has no explanatory power, it should be removed from theory in order not to risk its use as a contribution to violence myths.

Fourth, there are better explanations for violence than a “death drive.” Many studies have shown that similar violent acts were accompanied by various psychological content and motivations, that such acts required (mental and other) preparations, tried to achieve conscious targets, and could be fully controlled, if some supportive social context was available (for more details see Collins, 2008; Hamm and Spaaij, 2017; Buchholz, 2019, 2022). We plead to open psychoanalytic theorizing to these alternative and sometimes superior approaches to the problem of violence.

## Rationale

A lot has been written about the so-called death drive from the psychoanalytic perspective and various ideas were proposed about it in order to seek a possible clinical and developmental importance. However, there was not a single attempt to study the problem with the use of scientific methodology. Following Freud’s own idea that drives must be related to the hormones, we decided to gather and review biochemical evidence about the possible biological foundations of the “death drive.” Given that the concept itself is loosely defined, we use data about masochism and Gambling Disorder as its hypothesized manifestations, which are at the same time well studied in neurophysiological and biochemical research.

### Biochemical research related to Freud’s general notions about the drives

Throughout its development, Freud’s theory of the motivational drives remained a dualistic one and focused on conflict (Holder, 1970). For Freud drives “represent an instigation to mental activity” (Freud, 1926, p. 200). A drive-dependent activation of those neuroanatomical areas that represents the so-called SEEKING Command System^4^ is fully in keeping with Freud’s view. In 1915, Freud offered the architecture of his drives: pressure or motor factor, aim, object and source (Freud, 1915, p. 122). Already in 1905, he had clarified the nature of the motor factor (for the sexual drive): “It seems probable, then, that special chemical substances are produced in the interstitial portion of the sex-glands; these are then taken up in the blood stream and cause particular parts of the central nervous system to be charged with sexual tension.” (Freud, 1905, p. 215). Freud was probably unable to use the term “hormone,” which would have been appropriate for this proclamation because this term was coined in that same year by Starling (1905, p. 340).^5^

Inspired by Freud’s view to respect hormones as principle driving force of his drives, it was possible to link motor factors with known hormones (Kirsch and Mertens, 2018; Kirsch and Buchholz, 2020). The (imperative) motor factors address (not only one but) a variety of brain areas that were important for a specific drive activity (Kirsch, 2019). The view that hormones are responsible for the onset of a drive is not in conflict with Freud’s claim that the “aim of an instinct is in every instance satisfaction” (Freud, 1915, p. 122). During satisfaction, all Freudian drives secrete the brain chemical 5-hydroxytryptamine (Kirsch and Mertens, 2018). This signal molecule exerts a stimulatory control over pituitary release of β-endorphin in human beings (Petraglia et al., 1987; Maes et al., 1996), and endorphins are known to induce contentment or even euphoria at higher levels (Roth-Deri et al., 2008; Charbogne et al., 2014; Veening and Barendregt, 2015). In fact, β-endorphin release (that indicates also drive termination) is reported during the execution of hunger, thirst, sleep, sexual drive and attachment (Pilozzi et al., 2021).^6^ Of course, the view that only one signal molecule, for example, β-endorphin, would maintain exclusively (human) satisfaction is a severe oversimplification.^7^ However, in this manuscript β-endorphin—that is able to maintain a general state of well-being and pleasure by occupying the so-called μ-receptor (Bodnar and Klein, 2005; Veening and Barendregt, 2015)—is classified as a reporter molecule for human satisfaction. Therefore, when a psychological impulse (essential drives and harmful disorders (*vide infra*)) is expected to induce satisfaction, it must induce the release of β-endorphin as biochemical cachet.

In order to answer Freud’s question of “What instincts should we suppose there are, and how many?” (1915, p. 123), we advocated three criteria for identifying a motivational drive (Kirsch and Mertens, 2018):

1. an imperative nature of the drive as a psychological criterion,
2. orchestration *via* the *lateral hypothalamus* as a neurobiological cachet, and,
3. a drive termination by means of the central release of 5-hydroxytryptamine as a biochemical attribute.

By using these criteria, we identified the sexual drive, thirst, hunger (in line with Freud’s prediction), and sleep as Freudian motivational drives because all these drives will achieve the release of β-endorphin during satisfaction *via* a 5-hydroxytryptamine-dependent cascade (Kirsch and Buchholz, 2020). The question arises whether other essential drives can additionally lead to the release of β-endorphin.

Recently, we reported that the mother-infant bond also led to the release of β-endorphin (Kirsch and Buchholz, 2020), and this essential impulse was classified as an attachment drive. As advocated principally by Tinbergen (1989, p. 208), we follow the view that the onset of a drive can be achieved in an unconscious manner *via* actions of hormones secreted from an excited organ (Freudian drive type) or in a conscious one (Bowlbyian drive type) *via* action of excited sensory nerves (Kirsch and Buchholz, 2020). The release of β-endorphin is presumably not restricted to the mother-infant tie because the efficacy of other attachment bonds (i.e., pair bonding and social pair bonding; Bales et al., 2021) is also related to the secretion of this endorphin (Panksepp et al., 1980; Henry, 1982; Machin and Dunbar, 2011; Kyte et al., 2020).^8^

Most importantly, Freud claimed also that *Eros* (partly) neutralized the claims of the “death drive,” thereby preserving life:” The libido has the task of making the destroying instinct innocuous…” (Freud, 1924, p. 163). This statement assumes that essential drives are either able to down-regulate or to prevent the onset of a destructive impulse. From a biochemical perspective, there are various possibilities in order to achieve such a goal for metabolic pathways but in order to prevent getting into the labyrinth of facilities, the view is limited to only one attractive option. An essential drive A can render a second essential drive B by producing an appropriate inhibitor. Recently, we reported (Kirsch and Buchholz, 2020) that an operating mother-infant bond produces intermediately (in the body of the infant) the hormone oxytocin that is able to temporarily down-regulate the claim of the hunger drive. Thus, the attachment drive produces a signal molecule that renders the action of the hunger drive thereby confirming Harlow’s classical observations that social bonding is (temporarily) more important than hunger (Harlow, 1958). Since an operating essential drive can obviously influence the activity of other essential drives, it should be possible that operating essential drives would limit the activity of harmful impulses as predicted by Freud. How can essential drives achieve this goal?

The processing of a metabolic pathway can be described as a cascade of biochemical metabolites and recently we have demonstrated that such a strategy can also be applied to psychological drives (Kirsch and Buchholz, 2020). In order not to be confused by an armada of metabolites such a cascade is viewed as a biochemical stream. The first metabolite is the “spring” of such a biochemical stream, i.e., in the case of Freudian drives the corresponding executing hormones (= motor factors). All other metabolites of such a cascade can be classified as down-streaming products. Our suggested reporter molecule for human satisfaction (i.e., β-endorphin) is a very late down-streaming product of both Freudian drives and the mother-infant tie (Kirsch and Buchholz, 2020). Such late down-streaming metabolites are often responsible for initiating the inhibition of a metabolic pathway (which is described in every textbook of biochemistry). Since all drives activate the command system SEEKING (Kirsch, 2019; Kirsch and Buchholz, 2020) and because SEEKING runs on dopamine (Panksepp and Biven, 2012; Johnson et al., 2022), β-endorphin must be able to render the activity of SEEKING (probably by decreasing the dopamine activity) when β-endorphin acts as a general, late down-streaming metabolite of all drives. In fact, β-endorphin renders (striatal and hypothalamic) dopaminergic activity (Peres-Cruet et al., 1979; van Loon et al., 1980) *via* increasing dopamine re-uptake by dopaminergic nerve terminals (George and van Loon, 1982). In principle, the essential drives would be able to neutralize the claim of the “death drive” if the latter would also release β-endorphin as a late down-streaming product. In other words, if Freud’s proclamation “The libido has the task of making the destroying instinct innocuous” was correct, both classes of impulse collections (essential drives and harmful impulses (*vide infra*)) must have the identical end-product inhibitor. Consequently, a “death drive” must be a β-endorphin releaser, if its onset should be prevented by the operating essential drives. The negative-side of such a mechanism with an identical end-product inhibitor for essential drives as well as destructive impulses would be the down-regulation of (an) essential drive(s) by an overshooting “death drive.”^9^

To conclude. We need to remember that essential, β-endorphin releasing drives (i.e., the constituents of Freud’s *Eros* construct plus sleep plus attachment drives) are supposed to be in opposition to the (proposed) “death drive.” A control of the latter by the former would be possible if both classes of drives had the identical end-product inhibitor (i.e., β-endorphin and presumably other endorphins). In other words, since β-endorphin is our reporter molecule for achieving satisfaction, essential drives as well as harmful impulses must be able to lead to the feelings of well-being (or even euphoric states) if Freud’s proclamation is correct. Because of this mechanism, an opposite action, for example, the down-regulation of essential drives by an overshooting “death drive,” would be possible.

### Biochemical findings related to two proposed manifestations of the “death drive”

In order to “test” Freud’s idea of the “death drive” and the hormones possibly related to it, we will now review the biochemical correlates of some mental disorders that are supposed to be connected to the “death drive,” Masochism Disorder and Gambling Disorder.^10^

#### Masochism

The term “masochism” was introduced by Krafft-Ebing (1886) and Krafft-Ebing (1984) to describe individuals searching for (humiliating) slave positions during consensual sexual activities, referring to the novel “*Venus im Pelz (Venus in Furs)*” by Sacher-Masoch (1870).

The *Diagnostic and Statistical Manual V* (DSM-V) defines Masochism Disorder as a status lasting for at least 6 months, consisting of “recurrent and intense sexual arousal from the act of being humiliated, beaten, bound, or otherwise made to suffer as manifested by fantasies, urge or behaviors” (American Psychiatric Association, 2013, p. 694). The persistence of masochism is incompletely evaluated but has a mean age of onset of 19.3 years (American Psychiatric Association, 2013, p. 694; Fedoroff, 2020, p. 163).

Freud discussed the term “masochism” for the first time in his “Three Essays on the Theory of Sexuality” (Freud, 1905d), and the final formulations appeared 20 years later in “The Economic Problem of Masochism” (Freud, 1924). In 1920, he started connecting masochism to what he termed the “death drive”: “Masochism, the turning round of the instinct upon the subject’s own ego, would in that case be a return to an earlier phase of the instinct’s history, a regression” (pp. 54–55). Most importantly for the present manuscript, Freud classified primal masochism as: “the death instinct which is operative in the organism—primal sadism—is identical with masochism” (Freud, 1924, p. 164).

Now the question arises whether Freud’s suggestion of a primal masochism can be supported by experimental data. First, it should be noted that (our suggested reporter molecule for human satisfaction) β-endorphin is additionally released in order to mediate pain control (Roth-Deri et al., 2008; Charbogne et al., 2014; Veening and Barendregt, 2015). However, since β-endorphin manages also pain control, the next question arises whether pain can really lead to pleasure. As expected tentatively, pain has a negative correlation to pleasure but *pain relief* produces pleasure (Watanabe and Narita, 2018), most likely by activating brain structures of the command system SEEKING (Navratilova and Porreca, 2014). By using these relationships, Henry concluded that “[t]his suggest that perhaps any pain, inflicted as in masochism, might raise endorphin levels with the result that at the time of orgasm the levels of endorphins would be higher than would result from the sexual activity alone.” (Henry, 1982, p. 235). In other words, the masochistic patients use *pain relief* to accelerate β-endorphin release during *orgasm*.

Now the question arises why Freud’s proclamation that “[t]he libido has the task of making the destroying instinct innocuous” failed in patients suffering from erotogenic masochism? Bowlby’s assistant, Ainsworth et al. (1978) identified three patterns of attachment (secure, anxious-resistant, anxious-avoidant) to which disorganized was added later on (Main and Solomon, 1986). Howell (2013, p. 234) noted that the anxious-resistant attachment pattern and the disorganized one “are the most underlying states of mind for many people with masochistic tendencies.”^11^ Thus, our prime example of a “death drive,” i.e., primal masochism, can operate effectively when an essential drive is corrupted.

An astute reader may now be interested in the answer why the activity of the essential drives (here especially attachment) is decreased in patients suffering from primal masochism? Howell noted additionally that many psychologically distinctive features of masochists (i.e., passivity, lack of will, hypnotic feelings of helplessness, and tendencies of revictimization) are also symptoms of traumatic stress (Howell, 2013, p. 234). From these observations the idea almost suggests itself, and the thought that trauma is responsible for the onset of masochism is actually widely supported by the community (Glenn, 1984; van der Kolk, 1989; Gabriel and Beratis, 1997; Gavin, 2010; Blum, 2012). Therefore, it would be safe to presume that the attachment drive is corrupted by a (childhood) trauma with the effect that the onset of the “death drive” can no longer be effectively counteracted by the essential drives.

Since there is now evidence that Freud’s “erotogenic masochism” can be classified as an addictive disorder (Kurt and Ronel, 2017; Klier and Winograd, 2019), and because Freud classified primal masochism as a “death drive,” it can be suggested that constituents of the construct “death drive” are special kinds of addictive disorders. The American Society of Addiction Medicine, A (2021) defines human addiction as “a treatable, chronic medical disease involving complex interactions among brain circuits, genetics, the environment, and an individual’s life experiences. People with addiction use substances or engage in behaviors that become compulsive and often continue despite harmful consequences.”

During the last decade, the Johnson research group collected evidence that healthy persons maintain in the central nervous system the concentrations of opioids (with β-endorphin as the most effective μ-receptor activator) to a characteristic range (Johnson and Faraone, 2013; Johnson et al., 2014; Johnson, 2018; Tabi et al., 2020; Anugu et al., 2021; Jackson et al., 2021). A persistent opioid maintenance leads to pathophysiological conditions, no matter whether it is very high levels [as is the case with some forms of autism (Anugu et al., 2021)] or very low ones (as is the case with consumers of opiates like heroin; (Johnson et al., 2014)). As outlined (*vide supra*), the essential drives (i.e., hunger, thirst, sexual drive, sleep, and attachment) provide β-endorphin during satisfaction. Since attachment is highly effective in releasing sufficient amounts of β-endorphin (Panksepp et al., 1980; Henry, 1982), the essential drives would lose their capabilities to maintain physiological opioid levels in the central nervous system after a childhood trauma dependent disturbance of attachment. Research has shown that even 91% of heroin addicts in treatment belong to insecure attachment patterns, compared to 45% in community samples (Grubač et al., 2011). Therefore, the solution statement of the masochistic patient to the problem of an unphysiological low opioid tone is to “boost” the β-endorphin release with pain relief during orgasm. In other words, the decreased release of β-endorphin by the (corrupted) essential drives can be (in part) compensated by the onset of an auto-addictive disorder, such as masochism.

From the outlined key-features of masochism, our tentative definition of a “death drive” has three characteristic building blocks:

1. A “death drive” is an auto-addictive disorder and cannot be attributed to intoxication.^12^
2. A “death drive” can be established when a psychological trauma has corrupted essential drives, especially attachment, so that the physiological release of β-endorphin is limited.
3. A “death drive” releases β-endorphin in a pathophysiological manner in order to compensate the deficit introduced by corrupted essential drive(s). From such a perspective, a “death drive” acts as a surrogate of an essential drive.

#### Gambling disorder

By using the above advocated criteria I to III, it should be possible to classify with confidence hitherto unmentioned auto-addictive disorders as forms of the “death drive.”

A very likely candidate for of a hitherto unmentioned form of the “death drive” is the so-called pathological gambling that was recently renamed as Gambling Disorder and is now grouped into the addictions category of the DSM-V (American Psychiatric Association, 2013). Since the gambling disorder is not initiated by abusing drugs (Clark et al., 2013; Keough et al., 2018), it can be classified as an auto-addictive disorder thereby addressing our suggested building block I (*vide supra*). Horak et al. (2021) found evidence that childhood trauma represented a significant predictor of a diagnosis of gambling disorder, in agreement with the suggestions of others (Felsher et al., 2010; Schwaninger et al., 2017). In line with our proposal (*vide supra*), the (childhood) trauma decreased here also the capabilities of attachment drives to release β-endorphin, because the onset of insecure attachment predicted the symptomatic expression of pathological gambling among adolescents (Magoon and Ingersoll, 2006; Terrone et al., 2021). It should be noted that other essential drives, for example, the hunger drive (Black et al., 2013) and the sexual drive (Grant and Steinberg, 2005; Cowie et al., 2019), are additionally often corrupted in patients suffering from gambling disorder. Thus, gambling disorder also fulfills the criterion II of our “death drive” classification. There are several pieces of evidence from neurobiological studies that gambling disorder leads to “reward” *via* the stimulation of dopamine networking (Zeeb et al., 2009; Clark et al., 2019). The involvement of β-endorphin is less well evaluated but there are several clinical trials with inhibitors of the μ-receptor during the course of gambling disorder (Grant and Hartman, 2008; Koob, 2009). From these studies, it can be concluded that gambling disorder is a β-endorphin releasing impulse thereby fulfilling all criteria for a “death drive.”

To conclude, a childhood trauma dependent disturbance of attachment can achieve the onset of an auto-addictive disorder but does not have to lead to the development of masochism because other artificial β-endorphin providers, like gambling disorder, can be established.

## Discussion and conclusion

The basic purpose of this paper is to review scientific evidence for several of Freud’s many claims about the so-called death drive. More specifically, we provide an overview of biochemistry findings related to (1) the possible opposition of “life drives” and “death drive,” and (2) masochism and gambling disorder as hypothesized manifestations of the “death drive.”

Freud’s idea of a “death drive” was initially very speculative and referred to cosmological, physical, psychological, and clinical dimensions. Over time, it was further developed by some psychoanalysts, and criticized, or indeed rejected, by others. Sadly, these two general attitudes—one that proneness to aggression is inborn, the other that it is a consequence of traumatization—were never fully scientifically explored (Dimitrijević, 2018). It seems logical that if such an inborn tendency exists, we should be able to find it “somewhere”—in human genes, tissues, hormones, neurotransmitters…

We followed the only reasonable hypothesis, proposed actually by Freud himself, which claims that the physiological correlate of drives must be hormones. Previous research has clearly highlighted the existence of mechanisms that lead from the satisfaction of hunger, thirst, sleep, sex, and attachment needs, to the secretion of 5-hydroxytryptamine, and, subsequently, β-endorphin – the reporter molecule for human satisfaction (Kirsch and Mertens, 2018; Kirsch, 2019; Kirsch and Buchholz, 2020). One could thus conclude that each of these satisfactions, or various combinations thereof, can be seen as independent drives that sometimes can be united. The logical next step now would be to look for the same or similar neurophysiological reaction to the satisfaction of the so-called “death drive.” To test this, we have reviewed research studies focused on the biochemical reactions related to two hypothesized derivatives of this “drive”: masochism and Gambling Disorder.

The currently existing body of biochemical research focused on these two conditions reveals that healthy human beings tend to establish a characteristic level of β-endorphin (and maybe also of other endorphins) in their brains. In principle, a physiological level of β-endorphin signals to the individual that everything is all right. Additional release of β-endorphin induces satisfaction (or well-being, or even euphoria). If the mechanism leading to the satisfaction of one of the essential drives (attachment needs, hunger, sexuality, sleep, and thirst) is corrupted, the β-endorphin supply becomes suboptimal and human beings begin to search for other sources of satisfaction (β-endorphin release). The two auto-addictive disorders under our scrutiny (masochism and Gambling Disorder) can also lead to the release of β-endorphin and compensate the deficit induced by the malfunctioning essential drives. In that sense, one can establish a pattern of masochism or pathological gambling because they lead to pleasure similar to the pleasure of the satisfaction of the essential drives.

It is, however, important to note, that the so-called “death drive” (now classified as a collection of auto-addictive disorders) cannot be omnipresent. For most people under regular circumstances, sexual masochistic activities or gambling in an amusement hall are nothing more than latent alternative options that will not be automatically activated when the essential drives are optimally satisfied. They only become active in a repetitive manner once the essential drives are persistently corrupted and become disorders when this frustration is chronic.

We believe that these findings can also lead to improvement of psychoanalysis as therapy. Most psychoanalysts would agree that a chronic “death drive” (i.e., masochism, pathological gambling, and maybe also Anorexia Nervosa) is often only marginally responsive to individual psychotherapy. We have claimed that “death drive” acts as a surrogate for impaired essential drive(s), and in nearly all the cases it is the attachment drive that is corrupted. Therefore, psychotherapeutic treatments should focus on revitalizing the impaired attachment drive by emphasizing interpersonal orientation and incorporating group psychotherapy into treatment plans.

We believe that this review should (and probably has to) lead to the revision of psychoanalytic drive theory in several important points:

1. There are several independent (drug-free) impulses that lead to β-endorphin dependent pleasure *via* a largely shared biochemical mechanism. To the best of current scientific knowledge, these include, on the one hand, attachment needs, hunger, sexuality, sleep, and thirst, and, on the other hand, auto-addictive disorders.
2. The essential drives seem to be to a large extent independent of one another and the universal hierarchical primacy of one of them (in Freud’s model—sexuality, in Melanie Klein’s—destructiveness) cannot be confirmed. As clinical and everyday social experiences show, even persons without mental or somatic disorders find it challenging to achieve balance between these drives and may exaggerate with satisfying one when others are frustrated (i.e., overeating due to loneliness or sexual frustration).
3. Although Freud spent decades trying to develop a model of conflict between two drives equally powerful and fundamental both chronologically and ontologically, the biochemical mechanisms related to what he termed “death drive” do not get active when the essential drives are satisfied, and consequently they cannot be seen as belonging to the same level of importance as the life drives. In other words, as long as we have emotionally available persons we will not searching for activities that lead to addiction.
4. The dynamic among the drives is also more complex than the psychoanalytic trauma models expect. Though the connection between trauma and the “death drive” is obvious, trauma seems to play the role of a trigger more than of a cause. Biochemical mechanisms are latently present in every brain but will not be activated unless there is a chronic external frustration that introduces dysfunction in the satisfaction of the essential drives. They seem to be always ready to jump from the bench and join the game, if one of the first team players gets injured for whatever reason, to use a metaphor related to sports. Thus, the so-called “death drives” are only surrogates of the essential drives.

Naturally, our study suffers from limitations and will hopefully inspire future efforts superior to this one:

1. In order to prevent getting into the labyrinth of biochemical possibilities, only well analyzed signal molecules, like β-endorphin, were advocated to have key importance for the processing of both essential drives and harmful impulses. Although such a strategy is supported by actual knowledge, our rationalizations might be prone to a revision when at present under-evaluated metabolites would have unexplored capabilities on our suggested signal-molecules. For instance, if endogenous cannabinoids would be able to regulate the μ-receptor, β-endorphin would lose its key importance for achieving satisfaction.
2. Although we (believe that we) have reviewed all available studies of the phenomena under scrutiny, their number is not very high and we are looking forward to further biochemical research in this domain.
3. Besides the already existing studies of the essential drives, there are other strong candidates whose biochemical mechanisms are still understudied. We hope researchers will turn their attention to curiosity, playfulness (also suggested in Solms, 2012, 2013, 2015), humor, generosity, possibly even aesthetic and religious feelings, as psychologist and even some psychoanalysts are speculating.
4. Since human aggression can be grouped in three independent parts (Meyer et al., 2016), it cannot be viewed as a “primary aggressive drive,” which may lead to conclusion that enhanced aggression is probably initiated by frustration (also suggested in Boag, 2014, 2017). However, more biochemical studies are necessary before we can fully understand this phenomenon or the related powerful sources of human motivation like narcissism, greed, jealousy, etc.
5. Our review suggests that the activation of the “death drive” is triggered by a traumatic cessation of satisfaction of the essential drives. Again, further research is needed that would elucidate how exactly this process unfolds.

Our review of the biochemical research of phenomena related to Freud’s notion of the “death drive” has, despite some limitations, brought forth important conclusions. It makes it very obvious that psychoanalytic drive theory must be fundamentally revised after important new scientific findings were gathered. The revision seems to be most necessary when it comes to:

1. admitting that the model was too simplified, because there are more than two drives operating simultaneously (hunger, thirst, sleep, sexual drive, attachment, and possibly many more still unidentified drives),
2. abandoning the idea of an independent “death drive,” which was in theory related to repetition compulsion and aggressiveness, while the evidence shows that it is activated if and only if the primary drives remain chronically unsatisfied.

## Data availability statement

The original contributions presented in the study are included in the article/supplementary material, further inquiries can be directed to the corresponding author.

## Author contributions

MK responsible for the biochemical part and the idea that Freuds death drive is a collection of auto-addictive diseases. At least an impact of 33%. AD responsible for the historical overview of psychoanalytic trauma theory and the key idea that a childhood trauma is mandatory for malfunctioning of essential drives. At least an impact of 33%. MB responsible for the overview between death drive and (social) violence. Introduced the key idea that a death drive cannot be an omnipresent psychologic entity. At least an impact of 33%. All authors contributed to the article and approved the submitted version.

## Conflict of interest

The authors declare that the research was conducted in the absence of any commercial or financial relationships that could be construed as a potential conflict of interest.

## Publisher’s note

All claims expressed in this article are solely those of the authors and do not necessarily represent those of their affiliated organizations, or those of the publisher, the editors and the reviewers. Any product that may be evaluated in this article, or claim that may be made by its manufacturer, is not guaranteed or endorsed by the publisher.

## Footnotes

^1^Ferenczi also confirmed sharing these ideas with Freud retrospectively (Ferenczi, 1926, p. 16; Freud to Ferenczi, 24 January 1932).

^2^Human aggression is much more complex than animal aggression and has been classified as "*acts that may lead to harm (toward self or others)*" (Meyer et al., 2016). Psychiatrists use three categories in order to define these aggressive acts: *psychotic*, *impulsive* and *predatory* (alternatively *organized*). The fact that human "aggression" represents a collection of independent motivations rules out the option that it can be classified as a primary drive. Interestingly, Meyer, Cummings, Proctor, and Stahl noted that persistent human aggression can be a consequence of traumatization.

^3^Panksepp´s (1998, 2018; Panksepp and Biven, 2012) so-called Command System PLAY is now classified as an instinct (Johnson et al., 2022).

^4^Neuropsychologist Jaak Panksepp (1998,^ 2018^; Panksepp and Biven, 2012; Solms and Panksepp, 2012) classifies seven different types of motivations that can evoke special behaviors, e.g., *seeking* for rewards/resources/sexual partners, *lust*, *caring*, and affection, loss and *panic*, *rage*, *fear*, and *play*. Special subcortical regions of the brain are involved with the processing to the corresponding emotions, which are classified as so-called Command Systems (labeled SEEKING, RAGE, FEAR, LUST, CARE, PANIC, and PLAY). According to Watt (2017) the following major, neuroanatomical structures were given as constituents of the SEEKING Command Systems: *ventral tegmental area, lateral hypothalamus, periaqueductal gray,* and *nucleus accumbens*.

^5^Hormones (motor factors in Freudian nomenclature) are responsible for the execution of drives. The general action of hormones can be explained by using the Lock-Key-Principle. Hormones will act as keys for their locks which are called hormone receptors or simply receptors. Whereas hormones can travel by using the blood stream, receptors are fixed on the surface of cells. The situation is now complicated by the fact that most hormones have typically more than one type of receptor. One receptor type is often fixed on the surface of the hormone releasing cells (so-called autocrine action). An occupation of this receptor is the signal for the cell to terminate the secretion of the hormone. The second, structurally different receptor is fixed on cells of the target organ and this endocrine action was mentioned by Freud with the brain as target organ for his proposed drives. An occupation of this receptor has different functions but the amplification of the hormone signal (e.g., neurotransmitter release) is quite common. The situation is now further complicated by the fact that most hormones have (sometimes different types of) receptors on different neurons and this make it is often hard to be sure which occupation is the most important one.

^6^In the case of the sexual drive β-endorphin manages both the termination of the drive (Pfaus and Gorzalka, 1987; Gessa et al., 1979; McIntosh et al., 1980; Fabbri et al., 1989) and the onset of euphoria (Miller and Baum, 1987). Similar findings related to β-endorphin were observed in the action of thirst (Evered, 1983; Yamamoto et al., 2000) and sleep (King et al., 1981; Przewlocka et al., 1986; Myer et al., 1990; Song et al., 2018). It is documented that social relationships increased β-endorphin in cerebrospinal fluids of monkeys (Martensz et al., 1986; Keverne et al., 1989) and that humans avoid social contacts at high β-endorphin levels as is evidenced by some forms of autism (Machin and Dunbar, 2011; Anugu et al., 2021) The situation is somewhat more complex when it comes to hunger. Hunger is initiated by the hormone ghrelin and terminated by the hormone leptin (Kirsch and Mertens, 2018; Kirsch, 2019, and references herein). Leptin can induce in the brain the release of β-endorphin (Cheung et al., 1997; Mercer et al., 2014) and β-endorphin is known to regulate food intake *via* a less-well defined pathway called "appetite" (Veening and Barendregt, 2015; Pilozzi et al., 2021). In conclusion, β-endorphin terminates the sexual drive, thirst, sleep and attachment but the hunger drive terminating hormone leptin produces β-endorphin in order to regulate appetite.

^7^Aerobic physical exercise, mindfulness meditation, yoga and similar activities yield also to well-being and contentment but these benefits are mainly evoked by endogenous cannabinoids (Dietrich and McDaniel, 2004; Hofmann et al., 2010; Fuss et al., 2015; Watkins, 2018; Siebers et al., 2021).

^8^Various other functions of β-endorphin in psychological situations are described in the works of Berridge (e.g., Smith and Berridge, 2007; Berridge, 2009; Berridge and Kringelbach, 2015; Castro et al., 2015).

^9^Remarkably, given that morphine and heroin are both agents that outcompete ß-endorphin on occupying μ-receptors and can therefore be classified as ß-endorphin-mimetic compounds, their consumers have a reduced activity of the essential drives, like hunger, attachment, and the sexual drive (Ringwood et al., 2021).

^10^A further "death drive" candidate may be the illness Anoxeria Nervosa because it is classified as an auto-addictive, β-endorphin-mediated disorder (Marrazzi and Luby, 1986) and it can be set on by a childhood trauma (Monteleone et al., 2014). However, the view that a childhood trauma is (more or less) a requirement for the onset of Anorexia Nervosa is at present not generally accepted.

^11^Similar findings exist in the case of many other mental disorders and the developmental and psychodynamic aspects of that have been elaborated (Dimitrijević, 2020, 2022).

^12^A "death drive" is characterized by an unphysiological, non-substance-related μ-receptor activation *via* artificially up-regulated *endogenous metabolites* (e.g., β-endorphin) in a repetitive manner thereby establishing an *auto-addictive process* which coincides with a relentless persistence. A *substance use disorder* is characterized by an unphysiological μ-receptor activation *via drug abuse* in a repetitive manner thereby establishing a *drug-mediated addictive process* which coincides with a relentless persistence.
